# Triglyceride-Glucose Index, Modifiable Lifestyle, and Risk of Colorectal Cancer: A Prospective Analysis of the Korean Genome and Epidemiology Study

**DOI:** 10.1007/s44197-024-00282-w

**Published:** 2024-08-05

**Authors:** Anthony Kityo, Sang-Ah Lee

**Affiliations:** 1 Department of Preventive Medicine, School of Medicine, Kangwon National University, 1 Gangwondeahakgil, Chuncheon, Gangwon, 24341 Republic of Korea; 2 Interdisciplinary Graduate Program in Medical Bigdata Convergence, Kangwon National University, 1 Gangwondeahakgil, Chuncheon, Gangwon, 24341 Republic of Korea

**Keywords:** Insulin resistance, Triglycerides, Fasting blood glucose, Diet, Obesity, Colorectal cancer

## Abstract

**Background:**

Insulin-mediated pathways plausibly explain the pathogenesis of colorectal cancer (CRC). The triglyceride-glucose index (TyG) is a surrogate of insulin resistance (IR), but its association with CRC in the Korean population has not been evaluated.

**Methods:**

From the 2004–2013 Korean Genome and Epidemiology Study, 98,800 participants aged 40–69 years were followed through 2020. Data on CRC incidence were obtained from the Korean National Cancer Center registry. Cox regression models and restricted cubic splines were fitted to examine the association between the TyG; *In [(triglycerides) × (fasting glucose)/2]* and CRC incidence. Joint effects of modifiable lifestyle factors and TyG on CRC risk were also investigated.

**Results:**

Median follow-up time was 10.6 years, and 699 CRC cases were observed. A unit-increment in TyG was associated with increased risk of CRC combined (hazard ratio, HR: 1.28, and 95% confidence interval, CI: 1.12–1.46), colon (1.29, 1.10–1.54), and rectal cancer (1.24, 1.01–1.52). Associations were dose-dependent, with linear associations observed for CRC and colon, but non-linear associations were observed for rectal cancer. A high TyG index (above 8.4) combined with overweight/obesity was linked to an increased risk of CRC (1.31, 1.07–1.61) and colon cancer (1.33, 1.03–1.72). When combined with low fruit and vegetable intake, the risks were higher for CRC (1.40, 1.12–1.74) and colon cancer (1.57, 1.18–2.09). Combined with high red meat consumption, the risks were elevated for CRC (1.32, 1.05–1.65) and colon cancer (1.52, 1.15–2.02).

**Conclusions:**

A high TyG index was associated with a higher risk of colorectal cancer, and the risk was highest among participants with a high BMI, low fruit and vegetable intake, and high intake of red meat, suggesting a role of both insulin resistance and modifiable lifestyle in colorectal cancer development.

**Supplementary Information:**

The online version contains supplementary material available at 10.1007/s44197-024-00282-w.

## Background

Digestive system cancers are significant contributors to the global cancer burden [1]. Specifically, colorectal cancer (CRC) is a highly frequent digestive tract cancer. By 2040, CRC incidence and mortality is projected to substantially increase across the globe [2]. Identifying drivers of CRC risk and changes in modifiable risk factors is acknowledged among preventive approaches for CRC [2]. Lifestyle factors such as overnutrition and obesity that stimulate insulin secretion are also risk factors for CRC [2–5]. Hyperinsulinemia and insulin resistance (IR) are common in overnutrition and obesity, which has led to the hypothesis that IR is related to cancer development [6].

Epidemiological studies have reported associations between IR and colorectal cancer [7–12]. Gold-standard techniques for measuring insulin sensitivity are expensive, time-consuming, and therefore difficult to implement in large-scale epidemiological studies in resource-limited settings [13]. Accordingly, studies on IR and CRC risk used the homeostasis model of insulin resistance (HOMA-IR), circulating C-peptide, or insulin-like growth factor 1 (IGF1-) as measures of IR and insulin production [7–12].

Surrogates of IR have been developed based on biochemical measurements routinely assessed in clinical settings. These include the triglyceride glucose index (TyG) computed as *ln [(triglycerides, mg/dl) × (fasting glucose, mg/dl)/2]* [14]. The TyG index is a simple, non-invasive surrogate marker that shows good prediction of IR measured by the glucose clamp technique, the gold standard technique for measuring IR [15]. Considering that the gold standard method is labor-and time-intensive, TyG is a more favorable marker of IR. [16]

Few epidemiological studies have evaluated the association between TyG index and CRC risk. Fritz and colleagues reported positive associations of TyG with obesity related gastrointestinal cancers including CRC [17]. A retrospective cohort study in Japan reported that TyG was associated with an increased risk of colorectal cancer [18]. To date, no large-scale prospective cohort study has comprehensively evaluated the TyG index in relation to CRC risk in the Korean population [17]. Using the Korean Genome and Epidemiology Study, we evaluated the association of the TyG index with CRC risk and examined the joint effects of a high TyG and selected lifestyle factors on CRC risk.

## Methods

### Study Participants

**Fig. 1:**
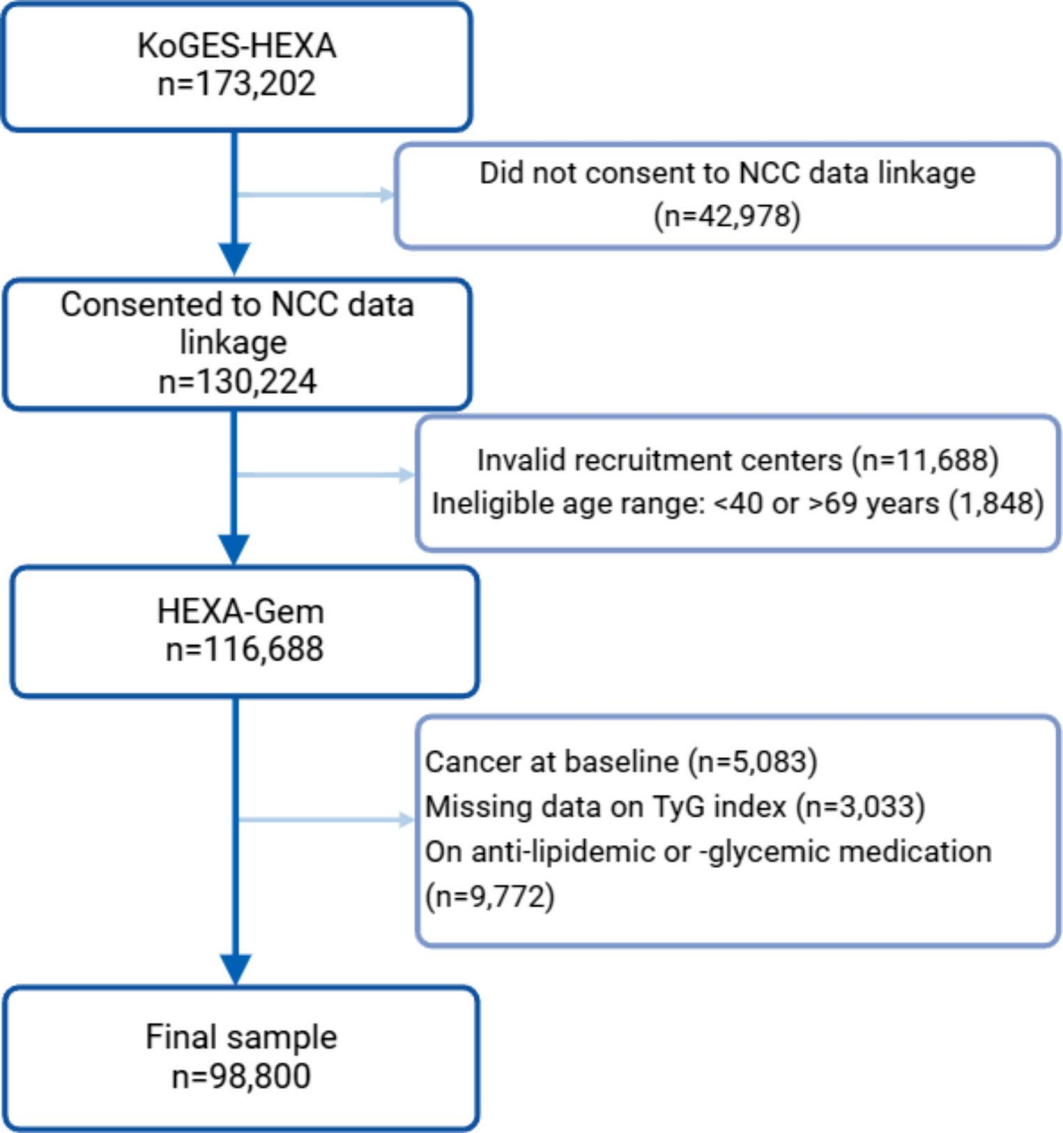
Illustration of the selection of study participants in the KoGES-HEXA cohort [21]

We derived an analytical sample from the Korean Genome and Epidemiology Study-Health Examinees cohort (KoGES-HEXA) of 173,202 participants who were recruited between 2004 and 2013 at 38 health examination centers and training hospitals located in eight regions of Korea [19, 20] and linked to the National Cancer Center (NCC) registry. After applying the exclusion criteria described in Fig. 1 and Supplementary Methods, 98,800 participants were analyzed.

### Exposure Variables

#### Biochemical Measurements

The methods used to process and measure biochemical parameters in the KoGES have been described elsewhere [22]. Baseline fasting plasma glucose and triglyceride levels were measured using enzymatic calorimetric methods with automatic analyzers (ADVIA 1650 and 1800; Siemens, Tarrytown, NY, USA). Using triglyceride and glucose measurements, the TyG index was calculated as In[(triglycerides, mg/dl) × (fasting glucose, mg/dl)/2] [14].

### Outcome Measures

Incident cases of CRC, colon, and rectal cancer were ascertained using the 10th revision of the International Classification of Diseases codes (ICD 10): colon (C18), rectum (C19-C21). Cases were ascertained from 2004 to December 31, 2020, via linkage to the NCC. The follow-up time for each participant was computed from the interview date to the date of cancer diagnosis, death, or until December 31, 2020, whichever occurred first.

### Assessment of Covariates

The KoGES used a standard interviewer-administered questionnaire to collect data on age, sex, educational level, household income, and lifestyle variables such as alcohol consumption, smoking, and physical exercise. Current alcohol drinkers were those who reported that they had consumed alcohol and were still drinkers alcohol at the time of the interview. Daily alcohol intake (g/day) was calculated for each participant using detailed alcohol assessment information from the questionnaire. Current smokers were defined as participants who had smoked > 400 cigarettes in their lifetime and were still smokers at the time of the interview [23]. Fruit, vegetable, and red meat intakes were computed from a semi-quantitative food frequency questionnaire and expressed as g/1000Kcal/day. Regular physical exercise was defined as engaging in activities that caused body sweating at least five times a week for at least 30 min per session. Height (m) and body weight (kg) were measured to the nearest 0.1 cm and 0.1 kg, respectively, while participants were wearing light clothes and barefooted. Body mass index (BMI) was calculated as body weight divided by height in meters squared (kg/m^2^).

### Statistical Analysis

For sample description, we categorized participants into fourths of TyG index. Missing data on income (8.7%) was assigned “unknown” while missing data on other categorical and continuous covariates (< 5%) was replaced by the mode and median respectively. We described participants’ characteristics according to fourths of the TyG using general linear models and chi-squared tests for continuous and categorical variables respectively. Continuous variables were described as mean (standard error), while categorical variables were described as percentages.

We useda SAS macro developed by Loic et al. [24] to construct restricted cubic splines (RCS) and examine the nonlinear association of TyG and CRC incidence, adjusting for age, sex, educational level, household income, alcohol consumption, smoking, physical exercise, BMI, fruit, vegetable, and total red meat intake. RCS were fitted with three knots at the 5th, 50th, and 95th percentiles of each index using the median of each index as a reference value. We then constructed Cox proportional hazard models and computed hazard ratios (HR) and 95% confidence intervals (CI) for a 1-unit increment in the TyG index adjusted for the same covariates as above, overall and stratified by sex.

We examined the joint effects of the TyG index (< median (8.4) and ≥ 8.4) and BMI (< 25.0 and ≥ 25.0 kg/m^2^), drinking (never/past drinkers and current drinkers); smoking (never/past smokers and current smokers); physical exercise (yes, no); fruit and vegetable intake, fiber intake, and total red meat intake (below and above median). We performed sensitivity analyses by excluding participants who were diagnosed with CRC in the first 2 years of follow to account for latent period bias/reverse causation. All analyses were performed using SAS software (version 9.4; SAS Institute Inc., Cary, NC, USA), and statistical significance was defined as *P* < 0.05.

## Results

Mean (SD) age was 53.2 (8.3) years. Participants contributed a total of 1,217,002 person-years (mean [SE]) of 10.6 (2.0) years, during which 699 CRC cancers (422 colon cancer cases) were observed. Participants with the highest TyG index were more likely to be older, male, middle school graduates, and low-income earners. A high TyG index positively correlated with current smoking, drinking, sedentary lifestyle, high BMI, and low fruit and vegetable intake (Table 1).

**Table 1:** Baseline characteristics of participants according to fourths of TyG index in the KoGES-HEXA cohort

	Quartiles of TyG index (*N* = 98,800)			
	Q1	Q2	Q3	Q4
n	24,705	24,696	24,706	24,693
Age, years, mean ± SE	50.2 ± 0.05	52.1 ± 0.05	53.3 ± 0.05	53.3 ± 0.05
BMI, kg/m^2^, mean ± SE	22.6 ± 0.02	23.4 ± 0.02	24.1 ± 0.02	25.0 ± 0.02
Sex, men, n (%)	4792 (19.4)	6742 (27.3)	8869 (35.9)	12,642 (51.2)
Education, up to middle school, n (%)	6065 (24.6)	7146 (28.9)	7976 (32.3)	7740 (31.3)
Monthly Income, < 3000 USD, n (%)	10,852 (43.9)	11,585 (46.9)	12,195 (49.4)	12,347 (50.0)
Current drinkers, n (%)	10,682 (43.2)	10,805 (43.8)	11,322 (45.8)	13,463 (54.5)
Current smokers, n (%)	2124 (8.6)	2938 (11.9)	3755 (15.2)	4968 (20.1)
Regular physical exercise, n (%)	9324 (37.7)	8614 (34.9)	8404 (34.0)	7729 (31.3)
BMI, ≥ 25.0 kg/m^2^, n (%)	3994 (16.2)	6369 (25.8)	8617 (34.9)	11,653 (47.2)
High blood glucose, n (%)	28 (0.1)	125 (0.5)	346 (1.4)	1927 (7.8)
Hypertriglyceridemia, n (%)	586 (2.4)	950 (3.9)	1383 (5.6)	2139 (8.7)
Triglycerides, mg/dL, mean ± SE	55.4 ± 0.37	86.6 ± 0.37	123.8 ± 0.37	229.8 ± 0.37
Fasting blood glucose, mg/dL, mean ± SE	86.3 ± 0.09	90 ± 0.09	93.1 ± 0.09	101.1 ± 0.09
TyG index, mean ± SE	7.7 ± 0	8.3 ± 0	8.6 ± 0	9.3 ± 0
Events, n				
Colorectal cancer	126	155	166	252
Colon cancer	68	101	102	151
Rectal cancer	58	54	64	101
Fruit and vegetable intake, g/1000 kcal/day, mean ± SE	177.45± 0.66	170.61± 0.66	164.81± 0.66	153.53± 0.66
Red meat intake, g/1000 kcal/day, mean ± SE	19.64± 0.11	19.26± 0.11	19.07± 0.11	20.31± 0.11

Values are expressed as percentages or means ± standard error

The HR and 95% CI for colorectal, colon, and rectal cancers per unit increase in the TyG index are shown in Table 2. TyG index was associated with an increased risk of colorectal (HR, 1.28; 95% CI, 1.12–1.46). colon (1.29, 1.10–1.54); and rectal (1.24, 1.01–1.52) cancers. The associations of TyG index and CRC risk persisted after excluding cases diagnosed within 2 years post-recruitment (Table 2).

**Table 2:** Hazard ratios and 95% CI of colorectal cancer risk per unit change in TyG index in the KoGES-HEXA cohort

	Events	Model ^1^	Model ^2^	Model ^3^	Model^2YR^	
		HR (95% CI)	HR (95% CI)	HR (95% CI)	*n*	
Colorectal	699	1.30 (1.15–1.47)	1.30 (1.14–1.48)	1.28 (1.12–1.46)	559	1.31 (1.14–1.52)
Colon	422	1.31 (1.11–1.54)	1.31 (1.11–1.54)	1.29 (1.10–1.54)	341	1.32 (1.10–1.59)
Rectum	277	1.29 (1.06–1.56)	1.29 (1.06–1.57)	1.24 (1.01–1.52)	218	1.30 (1.03–1.64)

Model ^1^: age and sex adjusted; Model ^2^: model 1 adjusted for educational level, monthly income, smoking, drinking, regular physical exercise; Model ^3^: model 2 adjusted for BMI, fruit and vegetable intake, and total red meat intake

Model^2YR^: excluding cases diagnosed within 2 years after index date

**Fig. 2:**
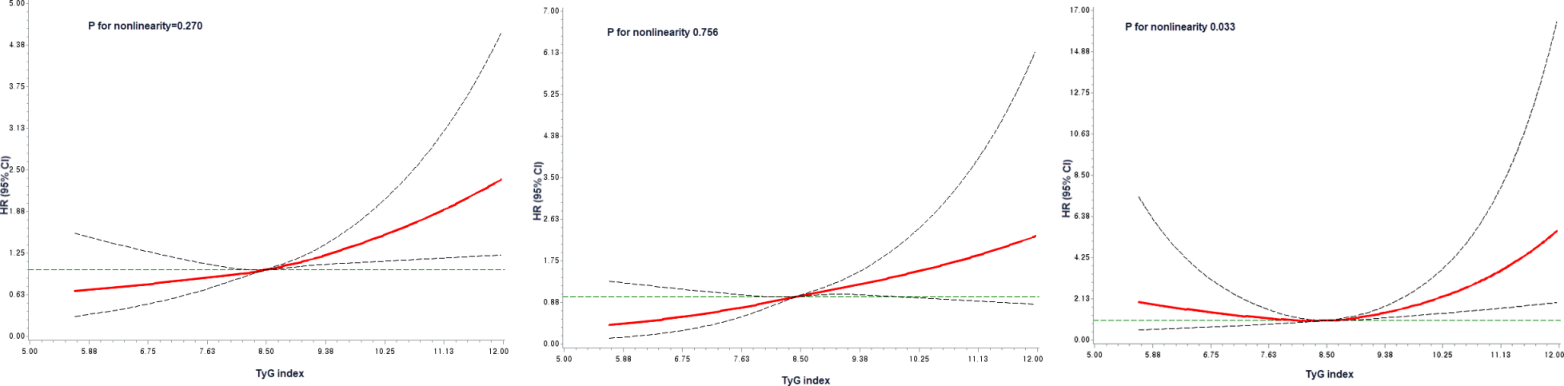
Dose-response associations between TyG index and colorectal cancer in the KoGES-HEXA cohort. The model was fitted using regression splines with three knots at the 5th, 50th, and 95th percentiles of TyG. The red line indicates hazard ratios (HR), and the dotted lines indicate the 95% CI. The model was adjusted for age, sex, educational level, monthly income, smoking, drinking, regular physical exercise, BMI, fruit and vegetable intake, and total red meat intake

Dose response analyses showed a linear increase in the risk of colorectal and colon cancer with increasing TyG index (*P* for nonlinear association, CRC, 0.27; colon, 0.756), but a non-linear association was observed for rectal cancer (*P* for nonlinear association = 0.033) (Fig. 2).

The association of TyG index with colorectal cancer incidence were generally consistent in men and women (Table 3).

**Table 3:** Hazard ratios and 95% CI of colorectal cancer risk per unit change in TyG index in the KoGES-HEXA cohort

	Men		Women	
	Events	HR (95% CI)	Events	HR (95% CI)
Colorectal	340	1.34 (1.12–1.60)	359	1.23 (1.01–1.49)
Colon	189	1.36 (1.05–1.40)	233	1.24 (0.98–1.58)
Rectum	151	1.30 (0.99–1.70)	126	1.19 (0.86–1.64)

Models were adjusted for age, sex, educational level, monthly income, smoking, drinking, regular physical exercise, BMI, fruit and vegetable intake, and total red meat intake

The joint associations of TyG and selected lifestyle factors with CRC incidence are shown in Table 4. Participants with overweight/obesity and high TyG levels had an elevated risk of CRC (HR, 95% CI: CRC: 1.31, 1.07–1.61; colon, 1.33, 1.03–1.72). Those with a high TyG index and low fruit and vegetable intake (CRC: 1.40, 1.12–1.74; colon, 1.57, 1.18–2.09) or high amounts of red meat (CRC: 1.32, 1.05–1.65; colon: 1.52, 1.15–2.02) had an increased risk of CRC compared to those with a low TyG index and high fruit and vegetable intake or low red meat intake (Table 4).

**Table 4:** Joint associations of TyG index and lifestyle factors with colorectal cancer risk in the KoGES-HEXA cohort

		*n*	Colorectal cancer				Colon cancer				Rectal cancer		
			< 8.4^a^	*n*	≥ 8.4	*n*	< 8.4	*n*	≥ 8.4	*n*	< 8.4	*n*	≥ 8.4
BMI, kg/m^2^	< 25.0	205	1.00	228	1.20 (0.99–1.45)	124	1.00	140	1.23 (0.96–1.58)	81	1.00	88	1.15 (0.85–1.57)
	≥ 25.0	74	1.09 (0.84–1.43)	192	1.31 (1.07–1.61)	43	1.05 (0.73–1.47)	115	1.33 (1.03–1.72)	31	1.16 (0.767–1.74)	77	1.28 (0.93–1.78)
Drinking	Never/past	140	1.00	201	1.30 (1.04–1.62)	94	1.00	125	1.21 (0.92–1.60)	46	1.00	76	1.47 (1.02–2.16)
	Current	139	1.24 (0.98–1.60)	219	1.35 (1.06–1.71)	73	1.05 (0.76–1.44)	130	1.34 (0.98–1.80)	66	1.61 (1.08–2.38)	89	1.40 (0.95–2.09)
Smoking	Never/past	231	1.00	320	1.19 (0.99–1.41)	143	1.00	198	1.21 (0.97–1.52)	88	1.00	122	1.15 (0.86–1.53)
	Current	48	0.98 (0.69–1.36)	100	1.17 (0.89–1.54)	24	0.86 (0.53–1.34)	57	1.19 (0.83–1.68)	24	1.15 (0.70–1.85)	43	1.14 (0.75–1.73)
Regular exercise	Yes	114	1.00	145	1.11 (0.87–1.42)	65	1.00	87	1.20 (0.86–1.67)	49	1.00	58	0.98 (0.66–1.45)
	No	165	0.89 (0.70–1.13)	275	1.10 (0.88–1.37)	102	0.97 (0.71–1.33)	168	1.23 (0.91–1.65)	63	0.78 (0.54–1.14)	107	0.93 (0.65–1.32)
Fruit and vegetable intake	≥Median	139	1.00	159	1.05 (0.83–1.33)	45	1.00	133	1.09 (0.81–1.48)	34	1.00	88	0.99 (0.68–1.43)
	< Median	140	1.07 (0.84–1.36)	261	1.40 (1.12–1.74)	122	1.15 (0.85–1.57)	122	1.57 (1.18–2.09)	78	0.96 (0.65–1.40)	77	1.15 (0.81–1.64)
Red meat intake	< Median	142	1.00	203	1.14 (0.92–1.43)	86	1.00	116	1.11 (0.83–1.47)	56	1.00	87	1.19 (0.84–1.69)
	≥Median	137	1.07 (0.84–1.36)	217	1.32 (1.05–1.65)	81	1.10 (0.81–1.49)	139	1.52 (1.15–2.02)	56	1.02 (0.70–1.49)	78	1.04 (0.72–1.49)

^a^TyG index dichotomized at the median value; Values are HR (95% CI)

Models were adjusted for age, sex, educational level, monthly income, smoking, drinking, regular physical exercise, BMI, fruit and vegetable intake, and total red meat intake

## Discussion

We investigated the association of TyG index and incidence of colorectal, colon and rectal cancers, and the joint effects with lifestyle factors. A high TyG index, meaning IR, was associated with about one-fourth to one-third increased risks of colorectal, colon, and rectal cancers, with linear associations observed for colorectal and rectal cancers, and non-linear association for rectal cancers. A high TyG index, together with overweight/obesity, low fruit intake, or high intake of red meat, jointly elevated the risk of colon cancer.

These findings provide additional epidemiological evidence of the role of IR in CRC carcinogenesis in diverse populations. Hyperinsulinemia and IR promote carcinogenesis via growth promoting and mitogenic effects of insulin and IGF-1 [4]. These ligands activate the 3-phosphorylated inositol-Akt-mammalian target of rapamycin (PI3-Akt-mTOR) and RAS-MAPK signaling pathways [28], which promote cell proliferation and survival, and protein and fatty acid synthesis [4, 16, 28, 29]. Hypertriglyceridemia and hyperglycemia are closely linked to hyperinsulinemia [30], and directly increase CRC risk by promoting fecal secondary bile acid secretion, altering insulin levels [31], and signaling pathways [30]. Elevated fecal secondary bile acids induce oxidative DNA damage, inflammation, NF-κB activation, and increase gut epithelial cell proliferation [32, 33].

The association of hyperinsulinemia and IR with CRC is consistent across studies and populations regardless of the IR measure used. IGF-1 levels were positively associated with CRC in the UK [10, 11], USA [8, 9], and Japan [12]. Plasma C-peptide levels were positively associated with CRC risk the USA and Japan [7, 9, 12]. In a Mendelian randomization study, fasting insulin was associated with an increased risk of CRC, but not of other cancers [34]. Using the TyG index, Fritz et al. reported an increased risk of colon and rectal cancers with a high TyG index in a European cohort [17]. Other studies have reported similar findings in Japan [18] and China [25, 26].

Lifestyle factors that stimulate insulin secretion, such as overweight/obesity, low fruit and vegetable intake, and high red meat consumption, are also risk factors for digestive tract cancers, especially CRC [3, 4]. Our study found that these factors exacerbated the positive association of IR and CRC, in line with their previous independent associations with an increased risk of CRC in the Korean population [35–37], and other populations [2, 38]. Our findings are supported by previous studies that reported synergistic effects of metabolic syndrome or insulin resistance and an unfavorable lifestyle on CRC incidence and prognosis [3, 39, 40]. Taken together, lifestyle modifications such as weight loss and optimal intake of fruits, vegetables, and red meat may be important for CRC prevention in individuals with IR. Indeed, large cohort studies have reported that favorable lifestyle patterns are associated with a substantial reduction in CRC risk [39, 41].

The current study is the first to evaluate the association of IR estimated from the TyG index, and the joint association of TyG and selected modifiable lifestyle with CRC risk in the Korean population. We used a large sample size, which enabled the evaluation of specific bowel cancers, employed a cohort study design which empowers causal inferences, and used standardized methods to measure both exposures and outcomes which enabled reduction of measurement errors. We also adjusted for known risk factors of CRC to reduce confounding. Nevertheless, we acknowledge some limitations, such as the use of single measurements of fasting blood glucose and triglycerides. However, these measurements have been shown to remain stable over time. In addition, although we included several covariates in our models, there is a possibility of residual unmeasured confounding.

## Conclusion

A high TyG index was associated with about one-fourth to one-third increased risk of colorectal, colon, and rectal cancers. Low fruit and vegetable intake, high BMI, and high intake of red meat heightened these risks, particularly for colon cancer. These findings highlight the role of IR in CRC development and suggest that weight management, optimal intake of fruits, vegetables, and red meat might improve CRC risk.

## Electronic Supplementary Material

Below is the link to the electronic supplementary material.

Supplementary Material 1

## Data Availability

Data from the Health Examinees (HEXA) study is part of the Korean Genome and Epidemiology Study (KoGES), conducted by Korea Disease Control and Prevention Agency (KDCA). The dataset analyzed in this study is maintained and managed by the Division of Population Health Research at the National Institute of Health, Korean Disease Control and Prevention Agency. It contains personal data that may potentially be sensitive to the patients, even though researchers are provided with an anonymized dataset that excludes resident registration numbers. Accordingly, the minimal data set used in the current study could not be publicly shared by the authors due to legal restriction on sharing sensitive patient information. To access the data, researchers are required to submit ethics approval, and a detailed research plan to the KDCA. Upon approval, the researchers are required to physically visit the KCDA and conduct the analysis from the KoGES data analysis room at the KCDA in Osong, Chungcheong Province, Republic of Korea. However, if the analysis does not involve linkage to the cancer registry, virtual access to the anonymized data set can be granted. Other researchers may request access to the anonymized data by contacting the following individuals at the Division of Population Health Research, National Institute of Health, Korea Disease Control and Prevention Agency: Senior Staff Scientist Dr. Jung Hyun Lee (jaylee1485@korea.kr); Director Dr. Kyoungho Lee (khlee3789@korea.kr).

## Abbreviations

BMI: Body mass index
CRC: Colorectal cancer
HEXA: Health Examinees Study
HOMAIR: Homeostasis model of insulin resistance
ICD 10: International Classification of Diseases codes 10th revision
IGF-1: Insulin-like growth factor 1
IR: Insulin resistance
KoGES: Korean Genome and Epidemiology study
PI3-Akt-mTOR: 3-phosphorylated inositol-Akt-mammalian target of rapamycin
RAS-MAPK: Ras-Mitogen-Activated Protein Kinase
RCS: Restricted cubic splines
TyG: Triglyceride-glucose index
