# Supplementary material for: Triglyceride-Glucose Index, Modifiable Lifestyle, and Risk of Colorectal Cancer: A Prospective Analysis of the Korean Genome and Epidemiology Study
Source: J Epidemiol Glob Health. 2024 Aug 5;14(3):1249–56. doi: 10.1007/s44197-024-00282-w (PMC11442717; doi:10.1007/s44197-024-00282-w)
Supplement: Supplementary file 1 — Supplementary Material 1 [file 44197_2024_282_MOESM1_ESM.docx]

**Supplementary Methods**

**Triglyceride-Glucose Index, Modifiable Lifestyle, and Risk of Colorectal Cancer: a Prospective Analysis of the Korean Genome and Epidemiology Study**

**Anthony Kityo, Sang-Ah Lee**

**Selection of Study participants**

Of the 173,202 participants, 42,978 did not consent to be linked to the National Cancer Center registry database and were excluded from the analysis. To yield to Health Examinees-Gem (HEXA-Gem) sub-sample, 11,688 participants from 1) sites that participated in the pilot study only from 2004 to 2006; 2) sites that did not meet the HEXA standards for biospecimen quality control; and 3) sites that participated in the study for < 2 years [21]; and 1848 participants aged < 40 years or > 69 years were excluded. There were 5,083 participants with cancer at baseline, 2 participants whose date of follow-up preceded the recruitment date, 3,033 with missing data on TyG index, and 9,772 participants on lipid- or glucose-lowering medication who were excluded from the HEXA-Gem sample, yielding a final analytical sample of 98,800 participants.
